# First-line treatment with sodium-glucose cotransporter 2 inhibitors and glucagon-like peptide-1 receptor agonists in type 2 diabetic population at low risk of cardiovascular disease: a meta-analysis

**DOI:** 10.3389/fendo.2024.1289643

**Published:** 2024-01-29

**Authors:** Rui Deng, Kaibo Mei, Tiangang Song, Jinyi Huang, Yifan Wu, Peng Yu, Zhiwei Yan, Xiao Liu

**Affiliations:** ^1^ Department of Operating Room, The Third Hospital of Nanchang, Nanchang, Jiangxi, China; ^2^ Department of Anesthesiology, The People’s Hospital of Shangrao, Shangrao, Jiangxi, China; ^3^ Department of Endocrinology, The Second Affiliated Hospital of Nanchang University, Nanchang, Jiangxi, China; ^4^ Department of Sports Rehabilitation, College of Human Kinesiology, Shenyang Sport University, Shenyang, China; ^5^ Department of Cardiology, Sun Yat-Sen Memorial Hospital of Sun Yat-Sen University, Guangdong Province Key Laboratory of Arrhythmia and Electrophysiology, Guangzhou, China

**Keywords:** sodium-glucose cotransporter 2 inhibitors, glucagon-like peptide-1 receptor agonists, type 2 diabetes, metformin, meta-analysis

## Abstract

**Background:**

The benefit of first-line use of sodium-dependent glucose transport 2 inhibitors (SGLT2i) and glucagon-like peptide-1 receptor agonists (GLP-1RAs) in type 2 diabetes mellitus (T2DM) with low risk of cardiovascular diseases are not clear.

**Methods:**

PubMed, EMBASE and Cochrane Library databases were searched to identify eligible randomized controlled trials. We used the odds ratio (OR) and mean difference (MD) and the corresponding 95% confidence interval (CI) to assess the dichotomous and continuous variable, respectively.

**Results:**

Thirteen studies involving 2,885 T2DM at low risk of cardiovascular diseases were included. Compared to placebo, first line use of SGLT2i significantly reduced glycosylated hemoglobin type A1C (HbA1c) (MD: -0.72), weight (MD: -1.32) and fasting plasma glucose (FPG) (MD: -27.05) levels. Compared with metformin, SGLT2i reduced body weight (MD: -1.50) and FPG (MD: -10.13) more effectively, with similar reduction for HbA1c (MD: -0.05). No significant increased safety adverse was found for SGLT2i, including nasopharyngitis (OR: 1.07), urinary tract infection (OR: 2.31), diarrhea (OR: 1.18) and hypoglycemia (OR: 1.06). GLP-1RAs significantly reduced HbA1c (MD: -1.13), weight (MD: -2.12) and FPG (MD: -31.44) levels as first-line therapy compared to placebo. GLP-1RAs significantly increased occurrence of diarrhea (OR: 2.18), hypoglycemia (OR: 3.10), vomiting (OR: 8.22), and nausea (OR: 4.41).

**Conclusion:**

First line use of SGLT2i and GLP-1RAs is effective in reducing HbA1c, weight, and FPG levels in T2DM patients at low risk for cardiovascular disease. SGLT2i may be superior to metformin in controlling body weight and FPG. GLP-1RAs may increase the occurrence of diarrhea, hypoglycemia, vomiting, and nausea.

**Systematic review registration:**

PROSPERO (International Prospective Register of Systematic Reviews. https://www.york.ac.uk/inst/crd, CRD42022347233).

## Introduction

The diabetes epidemic poses a significant economic and health burden for society and patients, with its prevalence increasing globally (1). Type 2 diabetes mellitus (T2DM) is the predominant type of diabetes and its global prevalence is projected to reach 439 million by 2030 (2). Recently, two novel antidiabetic drugs, sodium-glucose cotransporter 2 inhibitors (SGLT2i) and glucagon-like peptide 1 receptor agonists (GLP-1RAs), have been developed. Several large RCTs have shown convincing benefit of SGLT2i and GLP1-RAs in the glycemic control and reduction of cardiovascular events in patients with T2DM at high risk of CVDs. EMPA-REG OUTCOME (Empagliflozin, Cardiovascular Outcome Event Trial in Type 2 Diabetes Mellitus Patients) showed that Empagliflozin significantly reduced the composite of death from cardiovascular causes, nonfatal myocardial infarction, or nonfatal stroke in patients with T2DM patients at high cardiovascular risk (3). Another CANVAS Program (CANagliflozin cardio Vascular Assessment Study) trial also showed that canagliflozin significantly lowered cardiovascular events compared to placebo in T2DM patients at high risk cardiovascular diseases (4). REWIND (Researching Cardiovascular Events With a Weekly Incretin in Diabetes) illustrated that dulaglutide reduced body weight and primary composite outcome of composite endpoint of non-fatal myocardial infarction, non-fatal stroke, or death from cardiovascular causes in patients with T2DM and prior arteriosclerotic cardiovascular disease (ASCVD) or risk factors for ASCVD (5). However, there is some uncertainty regarding the efficacy of SGLT2i and GLP-1 RAs as first-line therapy for diabetic populations at low risk for cardiovascular disease. Therefore, the aim of this study was to investigate the efficacy and safety of SGLT2i and GLP-1RAs as first-line agents in treating patients with T2DM who are at a low risk for cardiovascular disease.

## Methods

The protocol of this study was prospectively registered with PROSPERO (International Prospective Register of Systematic Reviews. https://www.york.ac.uk/inst/crd, CRD42022347233). We followed a protocol for our systematic review and meta-analysis (**Supplementary S1, S2** for the updated study protocol and protocol deviations). The reporting items were followed by the Preferred Reporting Items for Systematic Reviews and Meta-Analyses (PRISMA) guidelines (**Supplementary Table 3**).

### Data sources and searches

Articles were searched regardless of region, publication type, language, or sample size. We searched PubMed, Embase, ClinicalTrails.gov and Cochrane Library for studies up to May 04, 2023. According to the PICOS (Population, Intervention, Comparison, Outcome and Study Design) principles, a literature search was conducted in conjunction with the following MeSH and keywords:

For population: ‘diabetes mellitus’ OR ‘diabetes’ OR ‘diabetic’.

For intervention and comparison: ‘SGLT-2 Inhibitor’ OR ‘SGLT-2 Inhibitors’ OR ‘Sodium-Glucose Transporter 2 Inhibitors’ OR ‘canagliflozin’ OR ‘empagliflozin’ OR ‘dapagliflozin’ OR ‘ipragliflozin’ OR ‘luseogliflozin’ OR ‘tofogliflozin’ OR ‘remogliflozin’ OR ‘sergliflozin’ OR ‘sotagliflozin’ OR ‘ertugliflozin’ OR ‘GLP-1 receptor agonist’ OR ‘GLP-1 receptor agonists’ OR ‘glucagon like peptide 1 receptor agonist’ OR ‘GLP-1 agonists’ OR ‘exenatide’ OR ‘liraglutide’ OR ‘albiglutide’ OR ‘lixisenatide’ OR ‘semaglutide’ OR ‘dulaglutide’ OR ‘taspoglutide’.

For outcome: No limitation.

For study design: Randomized Controlled Trials (RCT).

The detailed search strategy is described in **Supplementary Table 4**.

### Study selection

We developed eligibility criteria for inclusion in the literature based on the PICOS principles.

(1) Patients: adult patients with type 2 diabetes with low risk of cardiovascular diseases; not treated with antidiabetic medication or have not taken antidiabetic medication for a certain period of time; (2) intervention (exposure) and comparator: SGLT2 inhibitor or GLP-1 receptor agonist monotherapy versus placebo or metformin as a first-line drug; (3) outcomes: all clinical outcomes, such as cardiovascular event, death, changing in glycosylated hemoglobin type A1C (HbA1c), body weight, fasting plasma glucose (FPG) and safety outcomes. (4) study design: RCT published in full text. Exclusion of patients of the following cardiovascular diseases was defined as low risk of cardiovascular disease (such as myocardial infarction, heart failure, coronary heart disease, stroke, angina pectoris and other serious cardiovascular disease).

Studies reported effects of SGLT2i or GLP-1RAs in children or adolescents were excluded from the analysis. In cases where the same population was studied in multiple articles, data from the study with the largest number of experimental participants or the longest duration were used.

### Data extraction and risk of bias assessment

Two authors independently completed the literature search, data extraction, and quality evaluation of the included studies according to predetermined criteria. In case of disagreements, they were resolved by consensus. A third author reviewed the data.

The extracted data were as follows: (1) Name of the first author, publication year. (2) Study design (clinical trial registration number). (3) Participant characteristics included sample size, age and sex. (4) Interventions (control group/experimental group). (5) Follow-up duration. (6) Anti-diabetic drug history inclusion and exclusion criteria. (7) Outcomes.

Risk of Bias assessment of the included articles was conducted using the Risk of Bias version 2 (RoB2) tool (6) in the domains of the randomization process, deviations from intended interventions, missing outcome data, measurement of the outcome, and selection of the reported result.

### Statistical analyses

We performed all meta-analyses using RevMan (version 5.4, Cochrane Collaboration). The primary outcomes of the study were changes in HbA1c, body weight, and FPG from baseline, while safety outcomes were selected as secondary outcomes. Continuous variables were assessed using mean difference (MD) and corresponding 95% confidence intervals (CI), while dichotomous variables were assessed using combined odds ratio (OR) and corresponding 95% confidence intervals. The Cochrane Q test (p value and tau2) was used to assess heterogeneity among RCTs, with p values< 0.1 indicating statistical significance. A fixed-effects model was used when the heterogeneity was considered to be significant (7). I2 was used as a measure to assess the inconsistency across studies, and it was calculated using the formula $I^{2}=\frac{{\hat{\tau }}^{2}}{{\hat{\tau }}^{2}+{\hat{\sigma }}^{2}}$ , when ${\hat{\tau }}^{2}$ represented the sum of identical heterogeneity across all studies and ${\hat{\sigma }}^{2}$ represented the sum of sampling error across all studies (8). Publication bias test was not performed when the number of included reports was less than ten (9).

### Quality of evidence

We evaluated the quality and strength of the evidence for each outcome based on the Graded Recommendations Assessment, Development and Evaluation (GRADE) method. The evaluation was performed using GRADEprofiler software and two authors evaluated each outcome separately.

## Result

### Literature search

**Figure 1:**
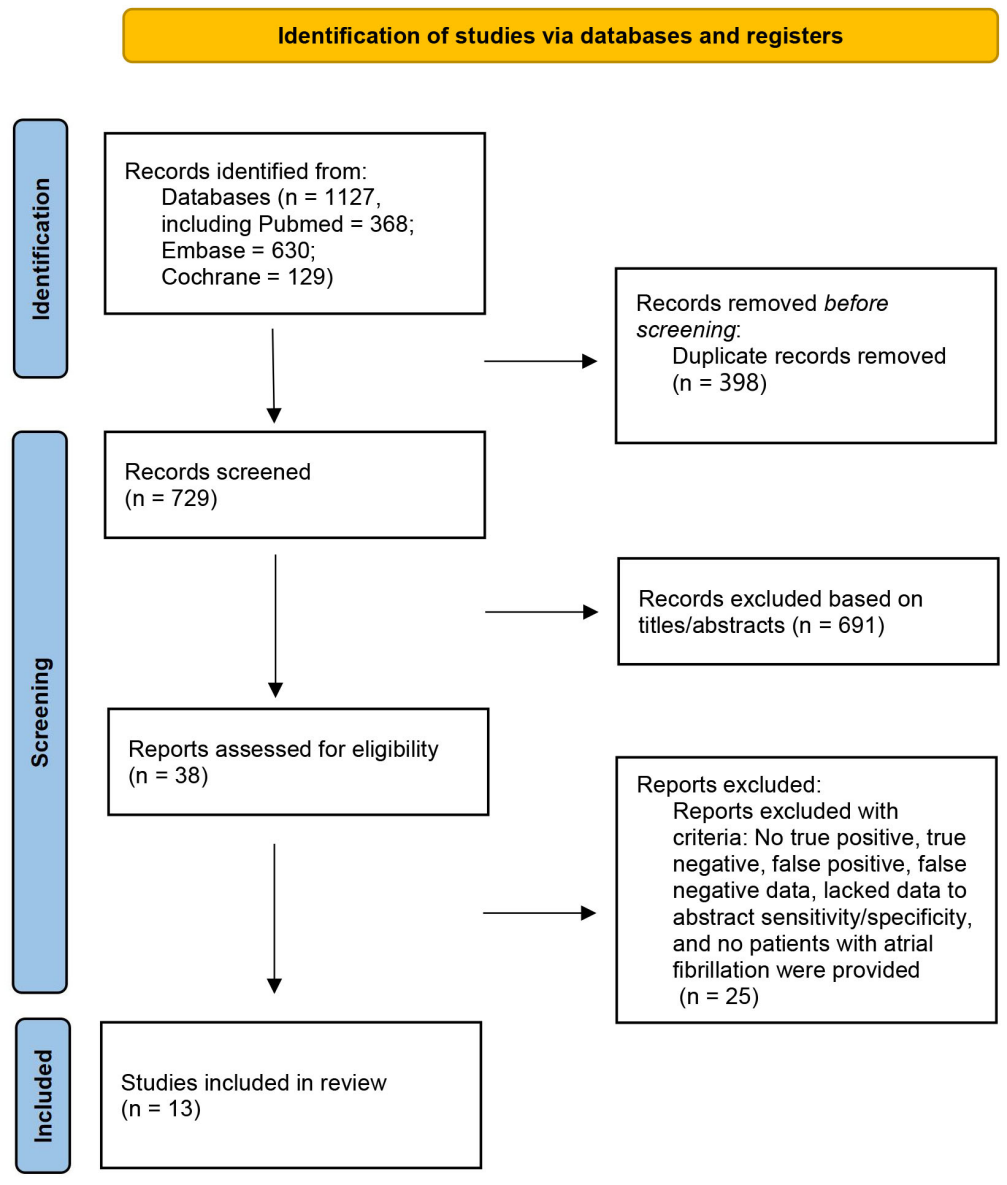
Flow chart of study selection in the meta-Analysis of first-line treatment with sodium-glucose cotransporter 2 inhibitors and glucagon-like peptide-1 receptor agonists in type 2 diabetic population at low cardiovascular disease risk.

After searching three databases, including PubMed, Embase, and Cochrane Library, a total of 1127 reports were identified (368 from PubMed, 630 from Embase, and 129 from Cochrane Library). After removing 398 duplicate articles, 691 articles were excluded based on screening of the titles and abstracts. Further, 38 articles underwent a more detailed full-text review. Finally, 13 RCT studies were deemed eligible and met the inclusion criteria (**Figure 1**) (10–22). All excluded studies and the reasons for their exclusion are shown in **Supplementary Table 5**.

### Study characteristics

**Table 1** provides an overview of the fundamental characteristics of all randomized controlled trials (RCTs) included in this study. These RCTs were published between 2008 and 2021, with a total of 2885 study subjects. Four studies (10, 11, 15, 19) were conducted to compare the efficacy of SGLT2i versus placebo, involving two drugs - Dapagliflozin, and Remogliflozin, with a total of 628 study subjects. Three studies (12, 13, 20) compared SGLT2i with metformin, with 923 subjects, and two drugs - Dapagliflozin and Canagliflozin. Six studies (14, 16–18, 21, 22) compared GLP-1RAs with metformin, involving four drugs - Semaglutide, Exenatide, Albiglutide, and Taspoglutide, with a total of 1,334 study subjects. The definition of first-line use and low risk for cardiovascular diseases varied across studies **Table 1** and **Supplementary Table 6**.

**Table 1:** Basic characteristics of the articles included in the meta-analysis.

First name, year, region	Sample size (C/I)	Control Intervention	Follow-up	Age (years) C/I	Male n (%) C/I	BMI (kg/m^2^) C/I	Definition for anti-diabetic drug naïve (first-line use)
Aroda 2019 (22), multi-center trial	353 (178/175)	Placebo/ Semaglutide 14 mg/day	26 weeks	54/54	89 (50.0)/86 (49.1)	32.2/31.7	No treatment with any medication for the indication of diabetes in a period of 90 days before the day of screening. An exception is short-term insulin treatment for acute illness for a total of below or equal to 14 days
Bailey 2012 (10), multi-center trial	136 (68/68)	Placebo/ Dapagliflozin 5 mg/day	24 weeks	53.5/51.3	37 (54.4)/32 (47.1)	32.47/30.97	Never having received medication or having received it for <24 weeks since the original diagnosis, no anti-hyperglycaemic therapy for >14 days during the 12 weeks prior to enrollment and no anti-hyperglycaemic therapy during the 4 weeks prior to enrollment
Ferrannini 2010 (11), multi-center trial	139 (75/64)	Placebo/ Dapagliflozin 5 mg/day	24 weeks	52.7/52.6	31 (41.3)/31 (48.4)	32.3/31.9	Drug naive, defined as never having received prescription medications for diabetes, having received prescription medications for diabetes for <24 weeks since the original diagnosis
Henry 2012 (12), multi-center trial	427 (208/219)	Metformin(+PBO)/ Dapagliflozin 10 mg(+PBO)/day	24 weeks	51.1/52.7	97 (46.6)/105 (47.9)	Not provided	Inclusion criteria: treatment naive males and females
Ito 2021 (13), Japan	21 (10/11)	metformin/ Dapagliflflozin 5 mg/day	12 weeks	57.5/55.9	8 (72.7)/9 (90)	26.7/27.7	Never received glucose-lowering agents
Ji 2014 (15), multi-center trial	260 (132/128)	Placebo/ Dapagliflozin 5 mg/day	24 weeks	49.9/53.0	87 (65.9)/84 (65.6)	25.93/25.17	Drug naive or treated with anti-diabetic medication for < 24 weeks
Ji 2021 (14), China	125 (62/63)	Placebo/ PB119 (exenatide) 200μg once weekly	12 weeks	50.7/51.4	37 (59.7)/41 (65.1)	Not provided	No treatment with any glucose lowing agent(s) within 3 months prior to screening.An exception is short-term treatment (no longer than 7 days in total) with insulin in connection with inter-current illness
Moretto 2008 (16), multi-center trial	155 (77/78)	Placebo/ Exenatide 10μg twice a day	24 weeks	53/55	42 (54.5)/48 (61.5)	32/31	Have not been treated with any antidiabetic agent
Nauck 2016 (17), multi-center trial	200 (101/99)	Placebo/ Albiglutide 50 mg once a week	52 weeks	53.1/52.0	58 (57.4)/50 (50.5)	33/33.9	Receiving no current antidiabetic therapy. The subject should not have received >7 contiguous days of any antidiabetic agent within the 3 months before Screening
Raz 2012 (18), multi-center trial	242 (123/129)	Placebo/ Taspoglutide 20mg once a week	24 weeks	55.8/55.0	43 (37)/46 (36)	Not provided	Drug naive patients with type 2 diabetes uncontrolled with diet and exercise;
Rosenstock 2016 (20), multi-center trial	475 (237/238)	Metformin/ Canagliflozin 300 mg/day	30 weeks	55.2/55.8	116 (48.9)/125 (52.5)	33.0/32.6	Not on antihyperglycemic agent therapy (at least 12 weeks before screening)
Sorli 2017 (21), multi-center trial	259 (129/120)	Placebo/ Semaglutide 1 mg once a week	30 weeks	53.9/52.7	70 (54)/80 (62)	32.40/33.92	No treatment with any glucose lowering agent(s) in a period of 90 days prior to screening. An exception is short-term treatment (no longer than 7 days in total) with insulin in connection with inter-current illness
Sykes 2015 (19), multi-center trial	93 (47/46)	Placebo/ Remogliflozin 50 mg twice daily	12 weeks	55.8/54.2	29 (62)/24 (52)	31.02/30.97	Subjects who are treatment-naïve and have not taken insulin, or any oral or injectable anti-diabetic medication in the past 3 months and have not taken a glucose lowering agent for ≥4 weeks at any time in the past, or Subjects who are newly diagnosed and treated with diet and exercise for a minimum of 6 weeks

C/I, Control group/Intervention group; BMI, body mass index.

## Main results

### HbA1c, body weight, and FPG

#### SGLT2i compared to placebo

**Figure 2:**
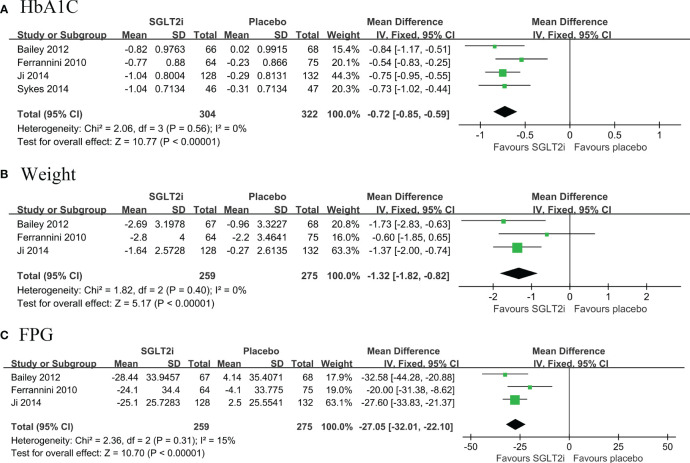
Forest plot of primary outcomes in SGLT2i and placebo groups. The diamond indicates the pooled estimate. Red boxes are relative to study size, and the black vertical lines indicate the 95% CIs around the effect size estimate. SGLT2i, sodium-glucose cotransporter 2 inhibitors. **(A)** HbA1C; **(B)** body weight; **(C)** Fasting Plasma Glucose.

Four RCTs were included, with a total of 626 patients (304 in the SGLT2i group and 322 in the placebo group), which compared the changes in both HbA1c and body weight among patients with T2DM and low cardiovascular disease risk, from baseline to follow-up (10, 11, 15, 19). Pooled results showed that SGLT2i as first line hypoglycemic agents significantly reduced HbA1c levels (MD: -0.72, 95% CI: -0.85, -0.59; I^2^ = 0%) (**Figure 2A**) and body weight levels with low heterogeneity (MD: -1.32, 95% CI: -1.82, -0.82; I^2^ = 0%) (**Figure 2B**). Pooled results from three RCTs (10, 11, 15) showed that SGLT2i significantly reduced FPG levels compared with placebo with low heterogeneity (MD: -27.05, 95% CI: -32.01, -22.10; I^2^ = 15%) (**Figure 2C**).

#### SGLT2i compared to metformin

**Figure 3:**
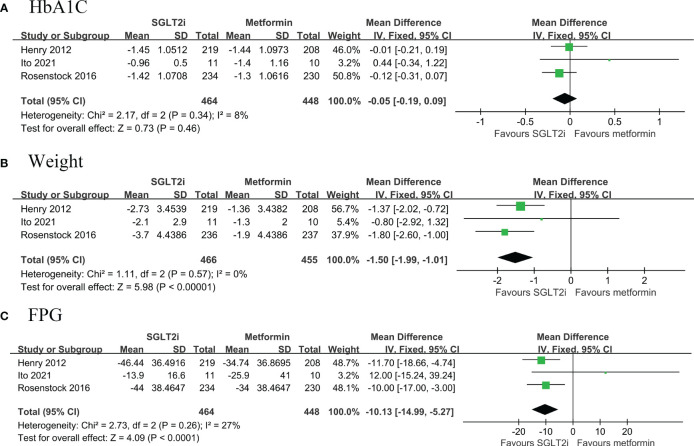
Forest plot of primary outcomes in GLP-1RAs and placebo groups. The diamond indicates the pooled estimate. Red boxes are relative to study size, and the black vertical lines indicate the 95% CIs around the effect size estimate. GLP-1RAs: glucagon-like peptide-1 receptor agonists. **(A)** HbA1C; **(B)** body weight; **(C)** Fasting Plasma Glucose.

A total of three RCTs (468 belonged to the SGLT2i group and 455 to the metformin group) compared the changes in HbA1c, body weight and FPG from baseline to follow-up in patients with T2DM (12, 13, 20). Pooled results showed that first line use of SGLT2i significantly reduced body weight (MD: -1.50, 95% CI: -1.99, -1.01; I^2^ = 0%) and FPG (MD: -10.13, 95% CI: -14.99, -5.27; I^2^ = 27%) compared with metformin, but not for HbA1c (MD: -0.05, 95% CI: -0.19, 0.09; I^2^ = 8%) (**Figures 3A–C**).

#### GLP-1RAs compared to placebo

**Figure 4:**
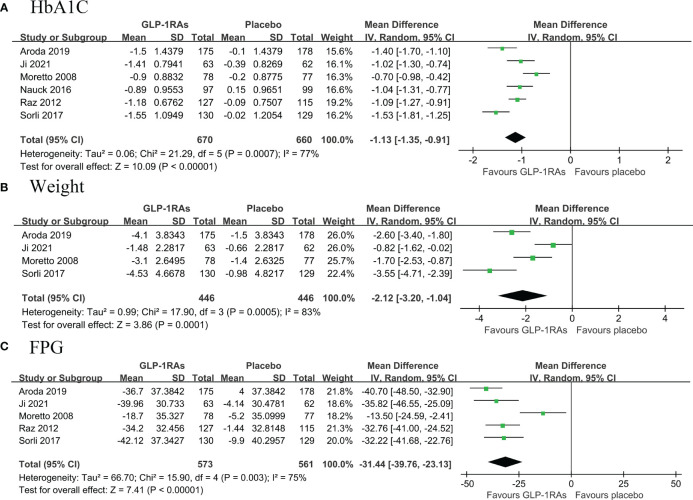
Forest plot of primary outcomes in SGLT2i and metformin groups. The diamond indicates the pooled estimate. Red boxes are relative to study size, and the black vertical lines indicate the 95% CIs around the effect size estimate. SGLT2i, sodium-glucose cotransporter 2 inhibitors. **(A)** HbA1C; **(B)** body weight; **(C)** Fasting Plasma Glucose.

A total of six RCTs (674 belonged to the GLP-1RAs group and 670 to the placebo group) compared the change in HbA1c from baseline to follow-up in patients with T2DM (14, 16–18, 21, 22). Pooled results showed that first line use of GLP-1RAs significantly reduced HbA1c levels with high heterogeneity (MD: -1.13, 95% CI: -1.35, -0.91; I^2^ = 77%) (**Figure 4A**). Pooled results from four RCTs (14, 16, 21, 22) showed that GLP-1RAs significantly reduced weight (MD: -2.12, 95% CI: -3.20, -1.04; I^2^ = 83%) and FPG levels (MD: -31.44, 95% CI: -39.76, -23.13; I^2^7 = 5%) compared with placebo with high heterogeneity. (**Figures 4B, C**).

### Safety outcomes: nasopharyngitis, urinary tract infection, diarrhea, hypoglycemia, vomiting, nausea

#### SGLT2i compared to placebo

Pooled results showed no significant differences between the first line use of SGLT2i and placebo for nasopharyngitis (OR: 1.07, 95% CI: 0.47, 2.42; I^2^ = 0%), urinary tract infection (OR: 2.31, 95% CI: 1.00, 5.34; I^2^ = 0%), diarrhea (OR: 1.18, 95% CI: 0.58, 2.39; I^2^ = 0%), or hypoglycemia in patients with T2DM (OR: 1.06, 95% CI: 0.37, 3.07; I^2^ = 0%). (**Supplementary Figure 1A**).

#### GLP-1RAs compared to placebo

In addition, the pooled results showed that first line use of GLP-1RAs has a significant predisposing effect on diarrhea (OR: 2.18, 95% CI: 1.32, 3.60; I^2^ = 10%), hypoglycemia (OR: 3.10, 95% CI: 1.34, 7.16; I^2^ = 0%), or vomiting (OR: 8.22, 95% CI: 4.02, 16.81; I^2^ = 24%). Similarity, pooled results found that GLP-1RAs increased nausea (OR: 4.41, 95% CI: 2.98, 6.55; I^2^ = 63%), but with high heterogeneity (**Supplementary Figure 1B**).

### Risk of bias assessment

Based on the RoB2 assessment, 64% of the included studies showed a low risk of bias. The intention-to-treat analysis had some concerns of bias in 36% of the studies. The detailed results of the RoB analysis are displayed in **Supplementary Figures 2A, 2B**.

### Publication bias and GRADE assessment

As per the guidelines, a public bias assessment was not conducted for this study due to the limited number of included studies. In the group that compared SGLT2i to placebo, the HbA1c estimates were of moderate certainty due to unclear selection bias results in two papers. The weight and safety estimates had moderate certainty because of the wide confidence interval. The FPG estimates had high certainty, as shown in **Supplementary Table 7A**.

In the group that compared SGLT2i to metformin, the HbA1c, weight, and FPG estimates had low certainty because of the unclear selection bias results and wide confidence intervals in the two studies, as shown in **Supplementary Table 7B**.

In the group that compared GLP-1RAs to placebo, the HbA1c, weight, and FPG estimates were rated as moderately certain due to high heterogeneity. The vomiting, nausea, and hypoglycemia estimates were of moderate certainty because of the wide confidence intervals. However, the diarrhea estimates were of low certainty due to high heterogeneity and wide confidence intervals, as indicated in **Supplementary Table 7C**.

## Discussion

### Main findings

In patients with T2DM and a low cardiovascular risk, first-line treatment with SGLT2i and GLP-1RAs led to significant reductions in HbA1c, body weight, and FPG levels. SGLT2i outperformed metformin in lowering body weight and FPG levels, but both were similarly effective in reducing HbA1c levels. Safety outcomes showed no significant difference between SGLT2i and placebo in nasopharyngitis, diarrhea, or hypoglycemia, albeit with a borderline increased risk of urinary tract infections. Conversely, GLP-1RAs increased the risk of diarrhea, hypoglycemia, vomiting, and nausea. To the best of our knowledge, this meta-analysis is the first to evaluate the effects and safety of SGLT2i and GLP-1RAs as first-line therapy for T2DM with low cardiovascular risk.

### Comparison with previous studies

The SUSTAIN 9 (Semaglutide once weekly as add-on to SGLT-2i therapy in T2DM) and SURPASS-1 (Efficacy and safety of a novel dual GIP and GLP-1 receptor agonist Tirzepatide in patients with type 2 diabetes) trials demonstrated that both SGLT2i and GLP-1RAs reduced HbA1c and body weight levels compared with placebo in patients with a history of medical treatment for diabetes (23, 24). Araki et al. found that empagliflozin had a stronger effect than metformin in lowering FPG and body weight, while their ability to lower HbA1c was similar (25). Fonseca et al. reported that a daily dose of 300 mg ipragliflozin and metformin was as effective as empagliflozin and metformin in reducing HbA1c, FPG, and body weight (26). However, a daily dose of 50 mg ipragliflozin and metformin was comparable only in reducing these parameters. The findings of our study suggest that SGLT2i and GLP-1RAs are effective first-line treatments for patients with T2DM who have a low cardiovascular disease risk. Specifically, significant reductions in HbA1c, body weight, and FPG levels were observed with the use of these drugs. The efficacy of SGLT2i in reducing HbA1c was similar to that of metformin, but it was superior in reducing body weight and FPG. However, given the limited number of studies included in this analysis, further randomized controlled trials are warranted to assess the efficacy of SGLT2i and metformin as first-line treatments in a T2DM population at low risk of cardiovascular disease. Our comparison revealed that the effects of SGLT2i and GLP-1RAs as first-line treatments in patients with T2DM at low cardiovascular disease risk were comparable to those observed in non-first-line treatment studies.

### Safety outcomes

The SUSTAIN-9 trial demonstrated an association between SGLT2i use and genital and urinary tract infections (23). A large population-based study also reported a threefold increased risk of genital infections associated with SGLT2i use, particularly in the elderly population (27). However, our study did find a borderline significant association between SGLT2i use and an increased risk of urinary tract infection (OR: 2.31, 95% CI: 1.00, 5.34). This result indicates a potential risk for urinary tract infection with first-line SGLT2i in T2DM patients at low risk of cardiovascular disease. Furthermore, SGLT2i has been associated with acute kidney injury, diabetic ketoacidosis, lower extremity amputation, and fracture, but our study lacked sufficient data to analyze these outcomes, and more studies are needed.

Discrepancies in the SGLT2i or GLP-1RA type and dosage across studies may have influenced the results. In the SGLT2i related outcomes, the trial by Sykes et al. employed Remogliflozin dose of 50 mg, while the other RCTs employed Dapagliflozin of 5 mg. Sensitivity analysis showed that the combined OR shifted from -0.72 [95% CI (-0.85, -0.59)] to -0.71 [95% CI (-0.86, -0.57)] upon excluding the Sykes et al. (19), indicating a stable result. Moreover, there was no observed evidence of heterogeneity in the SGLT2i pooled results. Regarding the GLP-RAs related results, the study encompassed five drug classes. All of these were human analogs, with the exception of Exenatide. Our sensitivity analysis conducted by the exclusion of the study involving Exenatide showed consistency with the main results (data not shown). An excellent report reviewed the GLP-1 RA head-to-head clinical studies and demonstrated that all GLP-1 RA agents are effective in reducing HA1C levels in patients with T2DM. However, differences exist in the extent of the effect on HA1C and weight. For example, the LEAD-6 trial showed that Liraglutide reduced HA1C more than exenatide twice daily, while the effects on overall weight loss were similar. Our previous studies also showed that human-like GLP-1 RAs are more effective in reducing the MACE in patients with T2DM and HF. Thus, different types and doses of GLP1 RAs may contribute to the results of heterogeneity. However, the limited numbers of studies preceded us performing a subgroup analysis, which calls for further studies.

Peptide InnOvatioN for the Early diabetEs tReatment (PIONEER) trial showed that GLP-1RAs caused an overall nausea rate of 15-20% and were associated with gastrointestinal adverse events, but these were temporary and dose-dependent (28). These results are in line with the findings of our study. However, the Semaglutide arm of the PIONEER8 trial was not associated with hypoglycemic events, which contradicts the results of our study. Additional studies are needed to confirm our results.

### Underlying mechanism

**Figure 5:**
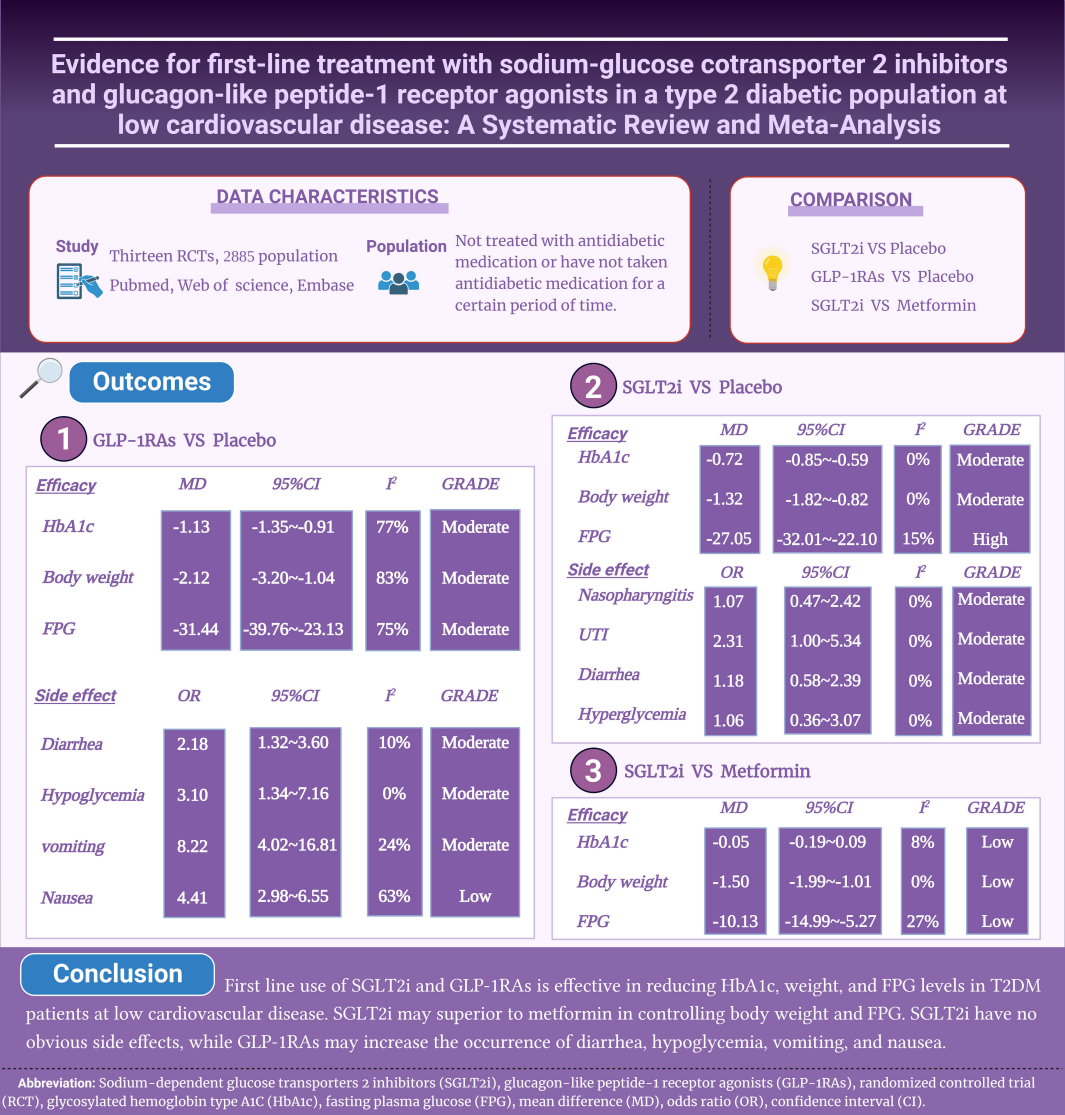
Mechanisms of SGLT2i and GLP-1RAs in patients with T2DM. GLP-1 receptor agonists, glucagon-like peptide-1 receptor agonists; SGLT2 inhibitors, sodium-glucose cotransporter 2 inhibitors; HbA1c, glycosylated hemoglobin type A1C; FPG, fasting plasma glucose.

The mechanisms that contribute to the beneficial effects of SGLT2i and GLP-1RAs in patients with T2DM have been extensively studied. (**Figure 5**) Briefly, SGLT2i inhibits the SGLT receptors in the proximal tubule, which inhibits the reabsorption of glucose from primary urine, resulting in glycosuria and a decrease in HbA1c and fasting glucose levels (29). The hypoglycemic effects of SGLT2i do not rely on insulin secretion, which minimizes the risk of hypoglycemia. Glycosuria-induced calorie loss leads to a reduction in subcutaneous and visceral adipose tissue, resulting in long-term or permanent weight loss (30). On the other hand, GLP-1RAs activate receptors in the pancreas, which stimulate glucose-dependent insulin secretion from pancreatic β-cells while inhibiting glucagon secretion from pancreatic α-cells (31). Additionally, GLP-1Ras stimulate GLP-1 receptors in the stomach, which inhibit gastric emptying, slowing gastrointestinal motility, and promoting satiety and decreased appetite, ultimately leading to weight loss (32). However, as evidenced in several studies and consistent with the results of this meta-analysis, adverse gastrointestinal events such as nausea, vomiting, and diarrhea have been reported in GLP-1RA experiments (33, 34). This adverse reaction is linked to gastrointestinal motility disturbances and delayed gastric emptying but is transient in duration (35).

### Clinical implications

Given the significant cardioprotective effect of SGLT2i and GLP-1RAs, the ADA guidelines state that all diabetic patients with confirmed or subclinical cardiovascular disease should be prescribed a GLP-1RA class or SGLT2i class with proven cardiovascular benefit, regardless of the patient’s HbA1c level or the presence of other glucose-lowering medications (36). SGLT2i and GLP-1RAs have been shown to have glucose-lowering effects in treated diabetic patients, and the current study demonstrates that they also have significant reductions in HbA1c, FPG, and weight in hypoglycemic drug naive patients with T2DM. The natural course of T2DM usually requires the use of multiple antihyperglycemic agents to achieve therapeutic goals, and the availability of new drugs has increased the diversity of drug regimens (37). Our study found that SGLT2i performed similarly to metformin in reducing HbA1c levels when used as first-line treatment for T2DM at low cardiovascular disease risk, but was more beneficial in reducing body weight and FPG levels with no significant side effects. Therefore, SGLT2i therapy could potentially be considered as first-line treatment for T2DM with low cardiovascular disease risk in the future. Moreover, a study demonstrated greater efficacy of a first-line use of SGLT2i compared with a first-line metformin strategy in terms of life expectancy and quality-adjusted life years (38). However, the high drug costs of first-line SGLT2i and GLP-1RAs render them cost-ineffective in the US population. A threshold analysis suggested that their costs would need to be reduced by at least 70% and 90% to reach a cost-effective willingness-to-pay threshold of $150,000 per quality-adjusted life-year (38). In addition, the safety outcomes of GLP-1RAs remain questionable. Large prospective trials are necessary to further validate the superior cardiovascular prognosis and safety of SGLT2i and GLP-1RAs to expand their applicability in diabetic patients with low cardiovascular disease risk.

### Strengths and limitations

To the best of our knowledge, this is the first meta-analysis to assess the effects of SGLT2i and GLP-1RAs as first-line therapeutic agents in T2DM patients with low cardiovascular disease risk. However, our meta-analysis does have some limitations. Firstly, although we defined our inclusion criteria for anti-diabetic drug-naïve patients as those who had not received any antidiabetic medication for at least three months (12 weeks), there might be some heterogeneity. Second, it is noteworthy that there was significant heterogeneity in patients with low cardiovascular disease risk (defined as those without severe cardiovascular diseases) across the included studies. Among the 14 trials included, four excluded patients with heart failure, while the remaining nine articles excluded patients with heart failure, cardiac events (e.g., myocardial infarction, unstable angina, revascularization procedure, stroke) or cardiovascular diseases (**Supplementary Table 4**). This heterogeneity precluded us from further interpreting the results and their generalizability. Generally, low cardiovascular risk is typically defined as the absence of cardiovascular disease and a Flemingham 10-year risk of cardiovascular events less than 20% (39, 40). Hence, it is evident that more comprehensive studies providing detailed cardiovascular risk data are necessary to achieve a more precise and refined grading of cardiovascular risk in future research concerning diabetes drugs.

## Conclusion

In conclusion, our meta-analysis showed that first-line use of SGLT2i and GLP-1RAs in T2DM patients at low cardiovascular disease risk significantly reduced HbA1c, body weight, and FPG levels in T2DM patients compared with placebo. Furthermore, SGLT2i outperforms metformin in lowering body weight and FPG levels. However, GLP-1RAs were associated with an increased incidence of diarrhea, hypoglycemia, vomiting, and nausea. Despite the positive results, due to the limited number of included studies, further randomized controlled trials are needed to investigate the safety and efficacy of SGLT2i and GLP-1RAs as first-line treatment options for patients with T2DM at low cardiovascular disease risk, particularly when compared to metformin.

## Data availability statement

The original contributions presented in the study are included in the article/**Supplementary Materials**, further inquiries can be directed to the corresponding author/s.

## Author contributions

RD: Conceptualization, Data curation, Formal analysis, Investigation, Methodology, Project administration, Resources, Software, Writing – original draft, Writing – review & editing. KM: Data curation, Formal analysis, Investigation, Methodology, Resources, Software, Visualization, Writing – original draft, Writing – review & editing. TS: Data curation, Formal analysis, Investigation, Methodology, Resources, Software, Visualization, Writing – original draft, Writing – review & editing. JH: Methodology, Software, Writing – review & editing. YW: Data curation, Resources, Software, Writing – review & editing. PY: Methodology, Software, Writing – original draft. ZWY: Conceptualization, Formal analysis, Supervision, Validation, Visualization, Writing – review & editing. XL: Conceptualization, Funding acquisition, Investigation, Project administration, Supervision, Validation, Visualization, Writing – original draft, Writing – review & editing.
