# Supplementary material for: First-line treatment with sodium-glucose cotransporter 2 inhibitors and glucagon-like peptide-1 receptor agonists in type 2 diabetic population at low risk of cardiovascular disease: a meta-analysis
Source: Front Endocrinol (Lausanne). 2024 Jan 29;15:1289643. doi: 10.3389/fendo.2024.1289643 (PMC10860745; doi:10.3389/fendo.2024.1289643)
Supplement: Supplementary file 1 [file DataSheet_1.docx]

**Electronic Supplementary Material**

**First-line treatment with sodium-glucose cotransporter 2 inhibitors and glucagon-like peptide-1 receptor agonists in type 2 diabetic population at low risk of cardiovascular disease: A Meta-Analysis**

Table S1. Updated Study protocol

Table S2. Protocol deviations from original study protocol

Table S3. PRISMA Checklist

Table S4: Search strategy

Table S5. Studies excluded (n=22) with reasons

Table S6. Specifics of the excluded cardiovascular disease population for each included literature

Table S7. GRADE assessment

Figure S1. Forest plot for the subgroup analysis

Figure S2. Cochrane Bias Risk Assessment Scale

Figure S3: Trial sequential analysis results

**SUPPLEMENTAL TABLES**

**Table S1.** Updated Study protocol

**Title**

First-line treatment with sodium-glucose cotransporter 2 inhibitors and glucagon-like peptide-1 receptor agonists in type 2 diabetic population at low risk of cardiovascular disease: A Meta-Analysis.

**Review question**

To investigate the efficacy and safety of SGLT2i and GLP-1RAs as first-line agents in treating patients with T2DM who are at low risk for cardiovascular disease.

**Searches**

EMBASE, PubMed, and Cochrane library from inception to from database inception to 22 July 2022 and updated on 04 May 2023 were performed.

**URL to search strategy.**

(Diabetes Mellitus OR diabetes OR pregnancy diabetes OR DM) AND (SGLT-2 Inhibitors OR Sodium-Glucose Transporter 2 Inhibitor OR Sodium-Glucose Transporter 2 Inhibitors OR canagliflozin OR empagliflozin OR dapagliflozin OR ipragliflozin OR luseogliflozin OR tofogliflozin OR remogliflozin OR sergliflozin OR sotagliflozin OR ertugliflozin OR GLP-1 agonist OR glucagon-like peptide-1 receptor agonist OR exenatide OR liraglutide OR albiglutide OR lixisenatide OR semaglutide OR dulaglutide OR taspoglutide ) AND (first-line treatment OR first-line therapy OR first-line drug OR first line)

**Condition or domain being studied.**

Type 2 diabetes combined with low cardiovascular disease risk.

**Participants/population**

adult patients with type 2 diabetes with low risk of cardiovascular diseases;

not treated with antidiabetic medication or have not taken antidiabetic medication for a certain period of time;

restrict the study population to cardiovascular disease (myocardial infarction or heart failure or coronary heart disease or stroke or angina pectoris or New York Heart Association;

Studies will be excluded as follows:

1) Replicated publications from the same study

2) Studies reported in children or adolescents

**Intervention(s), exposure(s)**

SGLT2 inhibitor or GLP-1 receptor agonist monotherapy versus placebo or metformin as a first-line drug.

**Comparator(s)/control**

Placebo or metformin as a first-line drug.

**Types of study to be included.**

RCT published in full text.

**Context**

**Main outcome(s)**

**1)** The primary outcome was effects of drugs (SGLT2i, GLP-1RAs, and metformin) on reducing HbA1C, body weight, and FPG2) The secondary outcomes were safety outcomes.

**2)** The secondary outcomes were safety outcomes.

**Measures of effect**

Pooled mean difference and odds ratio .

**Additional outcome(s).**

Not applicable.

**Data extraction (selection and coding)**

The following data will be independently extracted by two authors by two reviewers (ZY and TS) on an excel sheet

1. Name of the first author, publication year.

2) Study design (clinical trial registration number).

3) Participant characteristics included sample size, age and sex.

4) Interventions (control group/experimental group).

5) Follow-up duration.

6) Anti-diabetic drug history inclusion and exclusion criteria.

7) Outcomes.

**Risk of bias (quality) assessment**

Risk of Bias assessment of the included articles was conducted using the Risk of Bias version 2 (RoB2) tool in the domains of the randomization process, deviations from intended interventions, missing outcome data, measurement of the outcome, and selection of the reported result. Any discrepancies were handled by a senior investigator (XL).

**Strategy for data synthesis.**

All meta-analyses were conducted using RevMan (version 5.3; Cochrane Collaboration). The primary outcomes of the study were changes in HbA1c, body weight, and FPG from baseline, while safety outcomes were selected as secondary outcomes. Continuous variables were assessed using mean difference (MD) and corresponding 95% confidence intervals (CI), while dichotomous variables were assessed using combined odds ratio (OR) and corresponding 95% confidence intervals. The Cochrane Q test (p value and tau2) were used to assess heterogeneity among RCTs, with p values < 0.1 indicating statistical significance. A fixed-effects model was used when the heterogeneity was considered to be significant. I^2^ was used as a measure to assess the inconsistency across studies. Publication bias test was not performed when the number of included reports was less than ten.

**Analysis of subgroups or subsets.**

None.

**Type and method of review**

Meta-analysis, Systematic review

**Language**

No restriction

**Table S2.** Protocol deviations

| **Section** | **Previous Protocol** | **Publication** |
| --- | --- | --- |
| Title | SGLT-2 Inhibitors and GLP-1 Agonists: First-Line Therapy for Diabetes, A Meta-Analysis | First-line treatment with sodium-glucose cotransporter 2 inhibitors and glucagon-like peptide-1 receptor agonists in type 2 diabetic population at low risk of cardiovascular disease: A Meta-Analysis |
| Authors |  |  |
| Literature search | A literature search was performed using the electronic databases PubMed, EMBASE and the Cochrane Library from database inception to from inception to 14 July 2022 | EMBASE, PubMed, and Cochrane library from inception to from database inception to 22 July 2022 and updated on 04 May 2023 were performed. |
| Participants/population | Patients with type 2 diabetes who have not received antidiabetic treatment. | adult patients with type 2 diabetes with low risk of cardiovascular diseases;  not treated with antidiabetic medication or have not taken antidiabetic medication for a certain period of time;  restrict the study population to cardiovascular disease (myocardial infarction or heart failure or coronary heart disease or stroke or angina pectoris or New York Heart Association;  Studies will be excluded as follows:  1) Replicated publications from the same study  2) Studies reported in children or adolescents |
| Risk of bias appraisal | All selected papers will be assessed by two independent reviewers for methodological validity prior to inclusion in the review. The risk of bias will be evaluated using an adapted version of the Newcastle-Ottawa scale for cohort studies and using the Cochrane Risk of Bias Tool for the experimental trials. Discrepancies will be resolved by discussion with a third reviewer. Abstracts will automatically considered to be at high risk of bias because of the risk of abstract-to-publication discrepancies. | Risk of Bias assessment of the included articles was conducted using the Risk of Bias version 2 (RoB2) tool in the domains of the randomization process, deviations from intended interventions, missing outcome data, measurement of the outcome, and selection of the reported result. Any discrepancies were handled by a senior investigator (XL). |
| Strategy for data synthesis | Cochrane Risk of Bias Tool | the Risk of Bias version 2 (RoB2) tool |
| Strategy for data synthesis | The heterogeneity of the pooled hazard ratios was assessed using Cochran s Q test and Higgins I squared statistics. P less than 0.05 was considered statistically significant. For meta-analysis, a fixed-effects model and a random-effects model were considered for nonheterogeneous and heterogeneous data, respectively. Heterogeneity due to effect sizes across studies was assessed with the I 2 statistic. I values less than 25% indicated mild heterogeneity, I values between 25 and 50% indicated moderate heterogeneity, and I values more than 50% indicated large heterogeneity.17 The I values 50% or less were taken as indicators of the same scale of outcomes using a fixed-effects model. The I values more than 50% were taken as indicators of different scales of outcomes using a random-effects model, as recommended in the Cochrane Handbook for Systematic Review of Interventions. All analyses were performed with Review Manager 5.3. | All meta-analyses were conducted using RevMan (version 5.3; Cochrane Collaboration). The primary outcomes of the study were changes in HbA1c, body weight, and FPG from baseline, while safety outcomes were selected as secondary outcomes. Continuous variables were assessed using mean difference (MD) and corresponding 95% confidence intervals (CI), while dichotomous variables were assessed using combined odds ratio (OR) and corresponding 95% confidence intervals. The Cochrane Q test (p value and tau2) were used to assess heterogeneity among RCTs, with p values < 0.1 indicating statistical significance. A fixed-effects model was used when the heterogeneity was considered to be significant. I2 was used as a measure to assess the inconsistency across studies. Publication bias test was not performed when the number of included reports was less than ten. |

**Table S3. PRISMA Checklist.**

| **Section and Topic** | **Item #** | **Checklist item** | **Location where item is reported** |
| --- | --- | --- | --- |
| **TITLE** | | |  |
| Title | 1 | Identify the report as a systematic review. |  |
| **ABSTRACT** | | |  |
| Abstract | 2 | See the PRISMA 2020 for Abstracts checklist. |  |
| **INTRODUCTION** | | |  |
| Rationale | 3 | Describe the rationale for the review in the context of existing knowledge. |  |
| Objectives | 4 | Provide an explicit statement of the objective(s) or question(s) the review addresses. |  |
| **METHODS** | | |  |
| Eligibility criteria | 5 | Specify the inclusion and exclusion criteria for the review and how studies were grouped for the syntheses. |  |
| Information sources | 6 | Specify all databases, registers, websites, organisations, reference lists and other sources searched or consulted to identify studies. Specify the date when each source was last searched or consulted. |  |
| Search strategy | 7 | Present the full search strategies for all databases, registers and websites, including any filters and limits used. |  |
| Selection process | 8 | Specify the methods used to decide whether a study met the inclusion criteria of the review, including how many reviewers screened each record and each report retrieved, whether they worked independently, and if applicable, details of automation tools used in the process. |  |
| Data collection process | 9 | Specify the methods used to collect data from reports, including how many reviewers collected data from each report, whether they worked independently, any processes for obtaining or confirming data from study investigators, and if applicable, details of automation tools used in the process. |  |
| Data items | 10a | List and define all outcomes for which data were sought. Specify whether all results that were compatible with each outcome domain in each study were sought (e.g. for all measures, time points, analyses), and if not, the methods used to decide which results to collect. |  |
|  | 10b | List and define all other variables for which data were sought (e.g. participant and intervention characteristics, funding sources). Describe any assumptions made about any missing or unclear information. |  |
| Study risk of bias assessment | 11 | Specify the methods used to assess risk of bias in the included studies, including details of the tool(s) used, how many reviewers assessed each study and whether they worked independently, and if applicable, details of automation tools used in the process. |  |
| Effect measures | 12 | Specify for each outcome the effect measure(s) (e.g. risk ratio, mean difference) used in the synthesis or presentation of results. |  |
| Synthesis methods | 13a | Describe the processes used to decide which studies were eligible for each synthesis (e.g. tabulating the study intervention characteristics and comparing against the planned groups for each synthesis (item #5)). |  |
|  | 13b | Describe any methods required to prepare the data for presentation or synthesis, such as handling of missing summary statistics, or data conversions. |  |
|  | 13c | Describe any methods used to tabulate or visually display results of individual studies and syntheses. |  |
|  | 13d | Describe any methods used to synthesize results and provide a rationale for the choice(s). If meta-analysis was performed, describe the model(s), method(s) to identify the presence and extent of statistical heterogeneity, and software package(s) used. |  |
|  | 13e | Describe any methods used to explore possible causes of heterogeneity among study results (e.g. subgroup analysis, meta-regression). |  |
|  | 13f | Describe any sensitivity analyses conducted to assess robustness of the synthesized results. |  |
| Reporting bias assessment | 14 | Describe any methods used to assess risk of bias due to missing results in a synthesis (arising from reporting biases). |  |
| Certainty assessment | 15 | Describe any methods used to assess certainty (or confidence) in the body of evidence for an outcome. |  |
| **RESULTS** | | |  |
| Study selection | 16a | Describe the results of the search and selection process, from the number of records identified in the search to the number of studies included in the review, ideally using a flow diagram. |  |
|  | 16b | Cite studies that might appear to meet the inclusion criteria, but which were excluded, and explain why they were excluded. |  |
| Study characteristics | 17 | Cite each included study and present its characteristics. |  |
| Risk of bias in studies | 18 | Present assessments of risk of bias for each included study. |  |
| Results of individual studies | 19 | For all outcomes, present, for each study: (a) summary statistics for each group (where appropriate) and (b) an effect estimate and its precision (e.g. confidence/credible interval), ideally using structured tables or plots. |  |
| Results of syntheses | 20a | For each synthesis, briefly summarise the characteristics and risk of bias among contributing studies. |  |
|  | 20b | Present results of all statistical syntheses conducted. If meta-analysis was done, present for each the summary estimate and its precision (e.g. confidence/credible interval) and measures of statistical heterogeneity. If comparing groups, describe the direction of the effect. |  |
|  | 20c | Present results of all investigations of possible causes of heterogeneity among study results. |  |
|  | 20d | Present results of all sensitivity analyses conducted to assess the robustness of the synthesized results. |  |
| Reporting biases | 21 | Present assessments of risk of bias due to missing results (arising from reporting biases) for each synthesis assessed. |  |
| Certainty of evidence | 22 | Present assessments of certainty (or confidence) in the body of evidence for each outcome assessed. |  |
| **DISCUSSION** | | |  |
| Discussion | 23a | Provide a general interpretation of the results in the context of other evidence. |  |
|  | 23b | Discuss any limitations of the evidence included in the review. |  |
|  | 23c | Discuss any limitations of the review processes used. |  |
|  | 23d | Discuss implications of the results for practice, policy, and future research. |  |
| **OTHER INFORMATION** | | |  |
| Registration and protocol | 24a | Provide registration information for the review, including register name and registration number, or state that the review was not registered. |  |
|  | 24b | Indicate where the review protocol can be accessed, or state that a protocol was not prepared. |  |
|  | 24c | Describe and explain any amendments to information provided at registration or in the protocol. |  |
| Support | 25 | Describe sources of financial or non-financial support for the review, and the role of the funders or sponsors in the review. |  |
| Competing interests | 26 | Declare any competing interests of review authors. |  |
| Availability of data, code and other materials | 27 | Report which of the following are publicly available and where they can be found: template data collection forms; data extracted from included studies; data used for all analyses; analytic code; any other materials used in the review. |  |

*From:*  Page MJ, McKenzie JE, Bossuyt PM, Boutron I, Hoffmann TC, Mulrow CD, et al. The PRISMA 2020 statement: an updated guideline for reporting systematic reviews. BMJ 2021;372:n71. doi: 10.1136/bmj.n71

**Table S4:** **Search strategy**

Detailed description of the search strategy

| **PubMed** | |
| --- | --- |
| #1 | (("Diabetes Mellitus"[Mesh]) OR (((diabetes) OR (diabetes mellitus)) OR (DM))) |
| #2 | ((((((((((((((SGLT-2 Inhibitors) OR (SGLT-2 Inhibitor)) OR (Sodium-Glucose Transporter 2 Inhibitor)) OR (Sodium-Glucose Transporter 2 Inhibitors)) OR (canagliflozin)) OR (empagliflozin)) OR (dapagliflozin)) OR (ipragliflozin)) OR (luseogliflozin)) OR (tofogliflozin)) OR (remogliflozin)) OR (sergliflozin)) OR (sotagliflozin)) OR (ertugliflozin)) |
| #3 | ((((((((GLP-1 agonist) OR (glucagon-like peptide-1 receptor agonist)) OR (exenatide)) OR (liraglutide)) OR (albiglutide)) OR (lixisenatide)) OR (semaglutide)) OR (dulaglutide)) OR (taspoglutide) |
| #4 | ((((first-line treatment) OR (first-line therapy)) OR (first-line drug)) OR (first line)) |
| #5 | #2 OR #3 |
| #6 | #1 AND #4 AND #5 |

| **Embase** | |
| --- | --- |
| #1 | 'diabetes mellitus'/exp OR 'diabetes' OR 'diabetic' |
| #2 | 'sodium glucose cotransporter 2 inhibitor'/exp OR 'SGLT-2 Inhibitor' OR 'SGLT-2 Inhibitors' OR 'Sodium-Glucose Transporter 2 Inhibitors' OR 'canagliflozin' OR 'empagliflozin' OR 'dapagliflozin' OR 'ipragliflozin' OR 'luseogliflozin' OR 'tofogliflozin' OR 'remogliflozin' OR 'sergliflozin' OR 'sotagliflozin' OR 'ertugliflozin' |
| #3 | 'glucagon like peptide 1 receptor agonist'/exp' OR 'GLP-1 receptor agonist' OR 'GLP-1 receptor agonists' OR 'glucagon like peptide 1 receptor agonists' OR 'GLP-1 agonists' OR 'exenatide' OR 'liraglutide' OR 'albiglutide' OR 'lixisenatide' OR 'semaglutide' OR 'dulaglutide' OR 'taspoglutide' |
| #4 | 'first line therapy'/exp OR 'first line treatment' OR 'first line' OR 'first line drug' |
| #5 | #2 OR #3 |
| #6 | #1 AND #4 AND #5 |

| **Cochrane** | |
| --- | --- |
| #1 | MeSH descriptor: [Diabetes Mellitus] explode all trees |
| #2 | 'diabetes' OR 'diabetic' |
| #3 | MeSH descriptor: [Sodium-Glucose Transporter 2 Inhibitors] explode all trees |
| #4 | 'SGLT-2 Inhibitor' OR 'SGLT-2 Inhibitors' OR 'Sodium-Glucose Transporter 2 Inhibitors' OR 'canagliflozin' OR 'empagliflozin' OR 'dapagliflozin' OR 'ipragliflozin' OR 'luseogliflozin' OR 'tofogliflozin' OR 'remogliflozin' OR 'sergliflozin' OR 'sotagliflozin' OR 'ertugliflozin' |
| #5 | 'GLP-1 receptor agonist' OR 'GLP-1 receptor agonists' OR 'glucagon like peptide 1 receptor agonist' OR 'GLP-1 agonists' OR 'exenatide' OR 'liraglutide' OR 'albiglutide' OR 'lixisenatide' OR 'semaglutide' OR 'dulaglutide' OR 'taspoglutide' |
| #6 | 'first line therapy' OR 'first line treatment' OR 'first line' OR 'first line drug' |
| #7 | #1 OR #2 |
| #8 | #3 OR #4 |
| #9 | #5 OR #8 |
| #10 | #6 AND #7 AND #9 |

**Table S5: Studies excluded (n=22) with reasons**

| **Studies excluded** | **Reasons** |
| --- | --- |
| Abid 2020[1] | Not the target outcome: Drug budgets and clinical costs. |
| Bailey 2015[2] | Not the target exposure: not monotherapy. |
| Ballav 2020[3] | Not the target participants: Type I dibetes. |
| Chen 2020[4] | Not RCT. |
| Choi 2022[5] | Not the target outcome: Drug budgets and clinical costs. |
| Deng 2019[6] | Do not meet the inclusion: Experimental population were antidiabetic drug naive or not receiving antidiabetic drugs for a long time or set for washout period. |
| Ferri 2015[7] | This is an abstract. |
| Ferrannini 2013[8] | Do not meet the inclusion: Experimental population were antidiabetic drug naive or not receiving antidiabetic drugs for a long time or set for washout period. |
| Fralick 2021[9] | Not RCT. |
| Gupta 2017[10] | Duplicated data from same population. |
| Haluzik 2018[11] | This is an abstract. |
| Ishii 2017[12] | Do not meet the inclusion: Experimental population were antidiabetic drug naive or not receiving antidiabetic drugs for a long time or set for washout period. |
| Kutoh 2016[13] | Not the target exposure: Lack of control group. |
| Kutoh 2018[14] | Not the target exposure: Lack of control group. |
| Li 2020[15] | Not the target exposure: Lack of control group. |
| Maegawa 2021[16] | Not the target exposure: Lack of control group. |
| Neeland 2016[17] | Do not meet the inclusion: Experimental population were antidiabetic drug naive or not receiving antidiabetic drugs for a long time or set for washout period. |
| Qiu 2016[18] | This is a meta analysis. |
| Seino 2008[19] | Without target data: did not provide the mean value. |
| Shin 2021[20] | Not RCT. |
| Tanimoto 2015[21] | Not the target exposure: DPP-4 and SGLT-2I. |
| Xing 2013[22] | Not the target outcome: The antiinflammation effect of exenatide in peripheral blood mononuclear cells. |
| Yaggi 2020[23] | Do not meet the inclusion: Experimental population were antidiabetic drug naive or not receiving antidiabetic drugs for a long time or set for washout period. |
| Roden 2013[24] | Do not meet the inclusion: There were no restrictions on cardiovascular disease in the included population. |
| Roden 2015[25] | Duplicated data from same population. |

1. Abid, M. and L. Abid, *PDB17 Budget IMPACT Analysis of Empagliflozin for the Treatment of Type 2 Diabetes Mellitus in Tunisia.* Value in Health, 2020. **23**: p. S508.

2. Bailey, C.J., et al., *Efficacy and safety of dapagliflozin monotherapy in people with Type 2 diabetes: A randomized double-blind placebo-controlled 102-week trial.* Diabetic Medicine, 2015. **32**(4): p. 531-541.

3. Ballav, C., et al., *Lixisenatide in type 1 diabetes: A randomised control trial of the effect of lixisenatide on post-meal glucose excursions and glucagon in type 1 diabetes patients.* Endocrinol Diabetes Metab, 2020. **3**(3): p. e00130.

4. Chen, T.H., et al., *Sodium-glucose cotransporter 2 inhibitor versus metformin as first-line therapy in patients with type 2 diabetes mellitus: a multi-institution database study.* Cardiovasc Diabetol, 2020. **19**(1): p. 189.

5. Choi, J.G., et al., *First-Line Therapy for Type 2 Diabetes With Sodium-Glucose Cotransporter-2 Inhibitors and Glucagon-Like Peptide-1 Receptor Agonists : A Cost-Effectiveness Study.* Ann Intern Med, 2022. **175**(10): p. 1392-1400.

6. Deng, H., et al., *Effect of baseline body mass index on glycemic control and weight change with exenatide monotherapy in Chinese drug-naïve type 2 diabetic patients.* Journal of Diabetes, 2019. **11**(7): p. 509-518.

7. Ferri, L. and E. Hardy, *Clinical trial results for exenatide once weekly: Summary of efficacy and safety data from eight randomized trials of 4330 patients with type 2 diabetes.* Endocrine Reviews, 2015. **36**.

8. Ferrannini, E., et al., *Phase IIb, randomized, placebo-controlled study of the SGLT2 inhibitor empagliflozin in patients with type 2 diabetes.* Diabetes, Obesity and Metabolism, 2013. **15**(8): p. 721-728.

9. Fralick, M., et al., *Comparative effectiveness and safety of sodium-glucose cotransporter-2 inhibitors versus metformin in patients with type 2 diabetes: An observational study using data from routine care.* Diabetes Obes Metab, 2021. **23**(10): p. 2320-2328.

10. Gupta, S., et al., *Long-term efficacy and safety of empagliflozin monotherapy in drug-naïve patients with type 2 diabetes in Indian subgroup: Results from a 76-week extension trial of phase III, double-blind, randomized study.* Indian Journal of Endocrinology and Metabolism, 2017. **21**(2): p. 286-292.

11. Haluzik, M., et al., *Effect and safety of oral semaglutide monotherapy in type 2 diabetes: PIONEER 1 trial.* Diabetologia, 2018. **61**: p. S20-S21.

12. Ishii, H., et al., *Improvement of quality of life through glycemic control by liraglutide, a GLP-1 analog, in insulin-naive patients with type 2 diabetes mellitus: the PAGE1 study.* Diabetol Metab Syndr, 2017. **9**: p. 3.

13. Kutoh, E., et al., *Ipragliflozin as an Initial Therapy in Drug Naïve Subjects with Type 2 Diabetes.* Drug Research, 2016. **66**(7): p. 345-350.

14. Kutoh, E., et al., *Two Glucose-Lowering Mechanisms of Canagliflozin Depending on Body Weight Changes in Drug-Naïve Subjects with Type 2 Diabetes.* Drugs R D, 2018. **18**(4): p. 309-315.

15. Li, Y.M., et al., *Efficacy and Safety of Dulaglutide Monotherapy Compared to Glimepiride in Oral Antihyperglycemic Medication-Naïve Chinese patients with Type 2 Diabetes: A Post Hoc Analysis of AWARD-CHN1.* Diabetes Ther, 2020. **11**(5): p. 1077-1090.

16. Maegawa, H., et al., *Real-world evidence for long-term safety and effectiveness of ipragliflozin in treatment-naïve versus non-naïve Japanese patients with type 2 diabetes mellitus: subgroup analysis of a 3-year post-marketing surveillance study (STELLA-LONG TERM).* Diabetol Int, 2021. **12**(4): p. 430-444.

17. Neeland, I.J., U. Salahuddin, and D.K. McGuire, *A safety evaluation of empagliflozin for the treatment of type 2 diabetes.* Expert Opinion on Drug Safety, 2016. **15**(3): p. 393-402.

18. Qiu, R., et al., *Canagliflozin: Efficacy and Safety in Combination with Metformin Alone or with Other Antihyperglycemic Agents in Type 2 Diabetes.* Diabetes Therapy, 2016. **7**(4): p. 659-678.

19. Seino, Y., et al., *Dose-dependent improvement in glycemia with once-daily liraglutide without hypoglycemia or weight gain: A double-blind, randomized, controlled trial in Japanese patients with type 2 diabetes.* Diabetes Res Clin Pract, 2008. **81**(2): p. 161-8.

20. Shin, H.J., et al., *Cardiovascular effectiveness of SGLT-2 inhibitors versus metformin as first-line treatment for type 2 diabetes.* Pharmacoepidemiology and Drug Safety, 2021. **30**(SUPPL 1): p. 42.

21. Tanimoto, M., et al., *Comparison of sitagliptin with nateglinide on postprandial glucose and related hormones in drug-naïve Japanese patients with type 2 diabetes mellitus: A pilot study.* Journal of Diabetes Investigation, 2015. **6**(5): p. 560-566.

22. Xing, X., et al., *Antiinflammation effect of exenatide in Chinese newly diagnosed and drug-naïve type 2 diabetic patients.* Diabetes, 2013. **62**: p. A262.

23. Yaggi, H.K., et al., *Obstructive sleep apnoe and cardiovascular, heart failure and mortality outcomes with empagliflozin versus placebo in the EMPA-REG OUTCOME trial.* European Heart Journal, 2020. **41**(SUPPL 2): p. 2883.

24. Roden, M., et al., *Empagliflozin monotherapy with sitagliptin as an active comparator in patients with type 2 diabetes: a randomised, double-blind, placebo-controlled, phase 3 trial.* Lancet Diabetes Endocrinol, 2013. **1**(3): p. 208-19.

25. Roden, M., et al., *Safety, tolerability and effects on cardiometabolic risk factors of empagliflozin monotherapy in drug-naïve patients with type 2 diabetes: a double-blind extension of a Phase III randomized controlled trial.* Cardiovasc Diabetol, 2015. **14**: p. 154.

**Table S6: Specifics of the excluded cardiovascular disease population for each included literature**

| **Studies** | **Exclusion criteria regarding cardiovascular disease** |
| --- | --- |
| Aroda 2019 | Any of the following: myocardial infarction, stroke or hospitalisation for unstable angina or transient ischaemic attack within the past 180 days prior to the day of screening and randomisation; Subjects presently classified as being in New York Heart Association Class III/IV. |
| Bailey 2012 | Patients were ineligible if they had been diagnosed with a cardiovascular disease or event within 6 months prior to enrollment. |
| Ferrannini 2010 | A cardiovascular event (including New York Heart Association class III/IV congestive heart failure) within 6 months of enrollment. |
| Henry 2012 | A cardiovascular event within 6 months or New York Heart Association Class III or IV congestive heart failure. |
| Ito 2021 | History of serious diabetic complications, findings suggestive of insulin dependency, heart failure, and history of myocardial or cerebral infarction. |
| Ji 2014 | Patients with currently unstable or serious cardiovascular, renal, hepatic, hematologic, oncologic, endocrine, psychiatric, or rheumatic diseases were also excluded. |
| Ji 2021 | A severe cardiovascular event within 6 months before screening. |
| Moretto 2008 | Had a history or presence of clinically significant cardiovascular disease within the year prior to inclusion in the study. |
| Nauck 2016 | CHF NYHA class III-IV. |
| Raz 2012 | Patients were excluded if they had significant complications associated with type 2 diabetes, history of bariatric surgery, pancreatic disease,cardiovascular disease within the past 6 months. |
| Rosenstock 2016 | Myocardial infarction, unstable angina, revascularization procedure, or cerebrovascular accident in 12 weeks before screening; history of New York Heart Association Functional Classification III and/or IV cardiac disease. |
| Sorli 2017 | Heart failure (New York Heart Association class IV), or any acute coronary or cerebrovascular events in the 90 days before randomisation. |
| Sykes 2014 | Current or history of significant comorbid diseases, such as cardiovascular, hepatic and renal conditions. |

**Table S7: GRADE assessments**

1. **SGLT2i and Placebo**

| **Quality assessment** | | | | | | | **No of patients** | | **Effect** | | **Quality** | **Importance** |
| --- | --- | --- | --- | --- | --- | --- | --- | --- | --- | --- | --- | --- |
|  |  |  |  |  |  |  |  |  |  |  |  |  |
| **No of studies** | **Design** | **Risk of bias** | **Inconsistency** | **Indirectness** | **Imprecision** | **Other considerations** | **SGLT2i** | **PBO** | **Relative (95% CI)** | **Absolute** |  |  |
| **HbA1c (Better indicated by lower values)** | | | | | | | | | | | | |
| 4 | randomised trials | serious^1^ | no serious inconsistency | no serious indirectness | no serious imprecision | none | 304 | 322 | - | MD 0.72 lower (0.85 to 0.59 lower) | ÅÅÅO MODERATE | CRITICAL |
| **weight (Better indicated by lower values)** | | | | | | | | | | | | |
| 3 | randomised trials | no serious risk of bias | no serious inconsistency | no serious indirectness | serious^2^ | none | 259 | 275 | - | MD 1.32 lower (1.82 to 0.82 lower) | ÅÅÅO MODERATE | IMPORTANT |
| **FPG (Better indicated by lower values)** | | | | | | | | | | | | |
| 3 | randomised trials | no serious risk of bias | no serious inconsistency | no serious indirectness | no serious imprecision | none | 259 | 275 | - | MD 27.05 lower (32.01 to 22.10 lower) | ÅÅÅÅ HIGH | IMPORTANT |
| **Nasopharyngitis** | | | | | | | | | | | | |
| 3 | randomised trials | no serious risk of bias | no serious inconsistency | no serious indirectness | serious^2^ | none | 12/260  (4.6%) | 12/275  (4.4%) | OR 1.07 (0.47 to 2.42) | 13 more per 1000 (from 18 fewer to 58 more) | ÅÅÅO MODERATE |  |
|  |  |  |  |  |  |  |  | 4.5% |  | 9 more per 1000 (from 12 fewer to 39 more) |  |  |
| **urinary tract infection** | | | | | | | | | | | | |
| 4 | randomised trials | no serious risk of bias | no serious inconsistency | no serious indirectness | serious^3^ | none | 17/306  (5.6%) | 8/322  (2.5%) | OR 2.31 (1.00 to 5.34) | 12 more per 1000 (from 14 fewer to 52 more) | ÅÅÅO MODERATE |  |
|  |  |  |  |  |  |  |  | 4% |  | 6 more per 1000 (from 7 fewer to 27 more) |  |  |
| **diarrhea** | | | | | | | | | | | | |
| 4 | randomised trials | no serious risk of bias | no serious inconsistency | no serious indirectness | serious^3^ | none | 17/462  (3.7%) | 15/483  (3.1%) | OR 1.18 (0.58 to 2.39) | 5 more per 1000 (from 13 fewer to 40 more) | ÅÅÅO MODERATE |  |
|  |  |  |  |  |  |  |  | 2.7% |  | 5 more per 1000 (from 11 fewer to 35 more) |  |  |
| **Hypoglycemia** | | | | | | | | | | | | |
| 3 | randomised trials | no serious risk of bias | no serious inconsistency | no serious indirectness | serious^2^ | none | 6/260  (2.3%) | 6/275  (2.2%) | OR 1.06 (0.36 to 3.07) | 1 more per 1000 (from 9 fewer to 26 more) | ÅÅÅO MODERATE |  |
|  |  |  |  |  |  |  |  | 2.2% |  | 1 more per 1000 (from 11 fewer to 29 more) |  |  |

^1^ The selection bias of the three studies was unclear
^2^ Confidence interval width
^3^ No explanation was provided

1. **GLP-1 RAs and Placebo**

| **Quality assessment** | | | | | | | **No of patients** | | **Effect** | | **Quality** | **Importance** |
| --- | --- | --- | --- | --- | --- | --- | --- | --- | --- | --- | --- | --- |
|  |  |  |  |  |  |  |  |  |  |  |  |  |
| **No of studies** | **Design** | **Risk of bias** | **Inconsistency** | **Indirectness** | **Imprecision** | **Other considerations** | **GLP** | **Placebo** | **Relative (95% CI)** | **Absolute** |  |  |
| **HbA1c (Better indicated by lower values)** | | | | | | | | | | | | |
| 6 | randomised trials | no serious risk of bias | serious^1^ | no serious indirectness | no serious imprecision | none | 670 | 660 | - | MD 1.13 lower (1.35 to 0.91 lower) | ÅÅÅO MODERATE | CRITICAL |
| **weight (Better indicated by lower values)** | | | | | | | | | | | | |
| 4 | randomised trials | no serious risk of bias | serious^1^ | no serious indirectness | no serious imprecision | none | 446 | 446 | - | MD 2.12 lower (3.2 to 1.04 lower) | ÅÅÅO MODERATE | IMPORTANT |
| **FPG (Better indicated by lower values)** | | | | | | | | | | | | |
| 5 | randomised trials | no serious risk of bias | serious^1^ | no serious indirectness | no serious imprecision | none | 573 | 561 | - | MD 32.66 lower (36.78 to 28.55 lower) | ÅÅÅO MODERATE | IMPORTANT |
| **vomiting** | | | | | | | | | | | | |
| 6 | randomised trials | no serious risk of bias | no serious inconsistency | no serious indirectness | serious^2^ | none | 64/674  (9.5%) | 7/670  (1%) | OR 8.22 (4.02 to 16.81) | 69 more per 1000 (from 30 more to 140 more) | ÅÅÅO MODERATE |  |
|  |  |  |  |  |  |  |  | 0.5% |  | 35 more per 1000 (from 15 more to 73 more) |  |  |
| **Nausea** | | | | | | | | | | | | |
| 6 | randomised trials | no serious risk of bias | no serious inconsistency | no serious indirectness | serious^2^ | none | 128/674  (19%) | 33/670  (4.9%) | OR 4.41 (2.98 to 6.55) | 137 more per 1000 (from 84 more to 204 more) | ÅÅÅO MODERATE |  |
|  |  |  |  |  |  |  |  | 4.8% |  | 134 more per 1000 (from 83 more to 200 more) |  |  |
| **Hypoglycemia** | | | | | | | | | | | | |
| 5 | randomised trials | no serious risk of bias | no serious inconsistency | no serious indirectness | serious^2^ | none | 22/544  (4%) | 7/541  (1.3%) | OR 3.1 (1.34 to 7.16) | 26 more per 1000 (from 4 more to 73 more) | ÅÅÅO MODERATE |  |
|  |  |  |  |  |  |  |  | 0.8% |  | 16 more per 1000 (from 3 more to 47 more) |  |  |
| **Diarrhea** | | | | | | | | | | | | |
| 5 | randomised trials | no serious risk of bias | serious^1^ | no serious indirectness | serious^3^ | none | 50/611  (8.2%) | 24/608  (3.9%) | OR 2.18 (1.32 to 3.6) | 43 more per 1000 (from 12 more to 89 more) | ÅÅOO LOW |  |
|  |  |  |  |  |  |  |  | 2.3% |  | 26 more per 1000 (from 7 more to 55 more) |  |  |

^1^ High heterogeneity
^2^ The confidence intervals are wide
^3^ No explanation was provided

1. **SGLT and Metformin**

| **Quality assessment** | | | | | | | **No of patients** | | **Effect** | | **Quality** | **Importance** |
| --- | --- | --- | --- | --- | --- | --- | --- | --- | --- | --- | --- | --- |
|  |  |  |  |  |  |  |  |  |  |  |  |  |
| **No of studies** | **Design** | **Risk of bias** | **Inconsistency** | **Indirectness** | **Imprecision** | **Other considerations** | **SGLT** | **MET** | **Relative (95% CI)** | **Absolute** |  |  |
| **HbA1c (Better indicated by lower values)** | | | | | | | | | | | | |
| 3 | randomised trials | serious^1^ | no serious inconsistency | no serious indirectness | serious^2^ | none | 464 | 448 | - | MD 0.05 lower (0.19 lower to 0.09 higher) | ÅÅOO LOW | CRITICAL |
| **weight (Better indicated by lower values)** | | | | | | | | | | | | |
| 3 | randomised trials | serious^3^ | no serious inconsistency | no serious indirectness | serious^2^ | none | 466 | 455 | - | MD 1.5 lower (1.99 to 1.01 lower) | ÅÅOO LOW | IMPORTANT |
| **FPG (Better indicated by lower values)** | | | | | | | | | | | | |
| 3 | randomised trials | serious^3^ | no serious inconsistency | no serious indirectness | serious^3^ | none | 464 | 448 | - | MD 10.13 lower (14.99 to 5.27 lower) | ÅÅOO LOW | IMPORTANT |

^1^ The selection bias of two studies was unclear
^2^ The confidence interval is wide
^3^ No explanation was provided

**SUPPLEMENTAL FIGURES**

**Figure S1** **Forest plot** **of safety outcomes**

1. **SGLT2i compared to the placebo group**

**
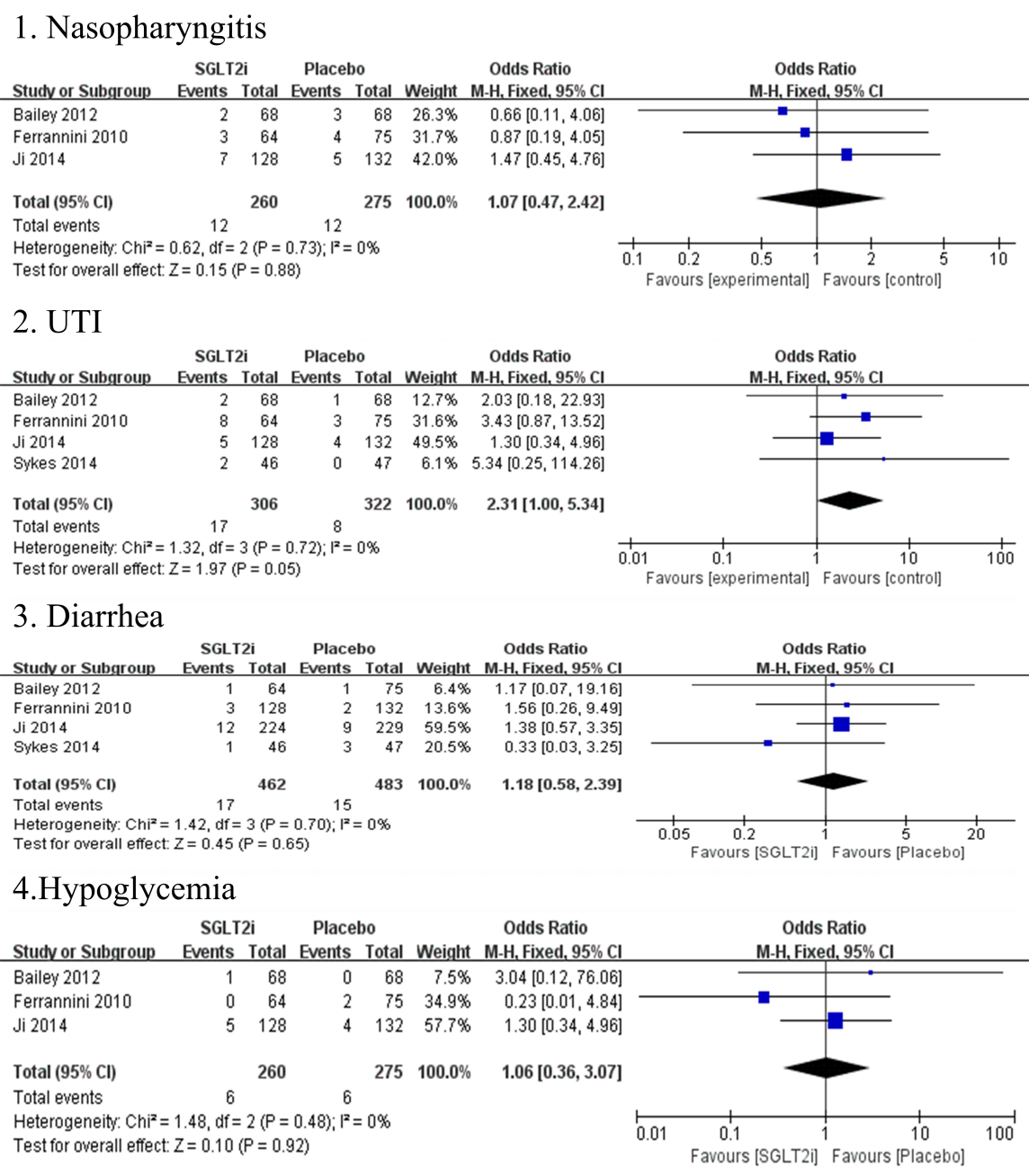
**

1. **GLP-1 RAs compared to the placebo group**

**
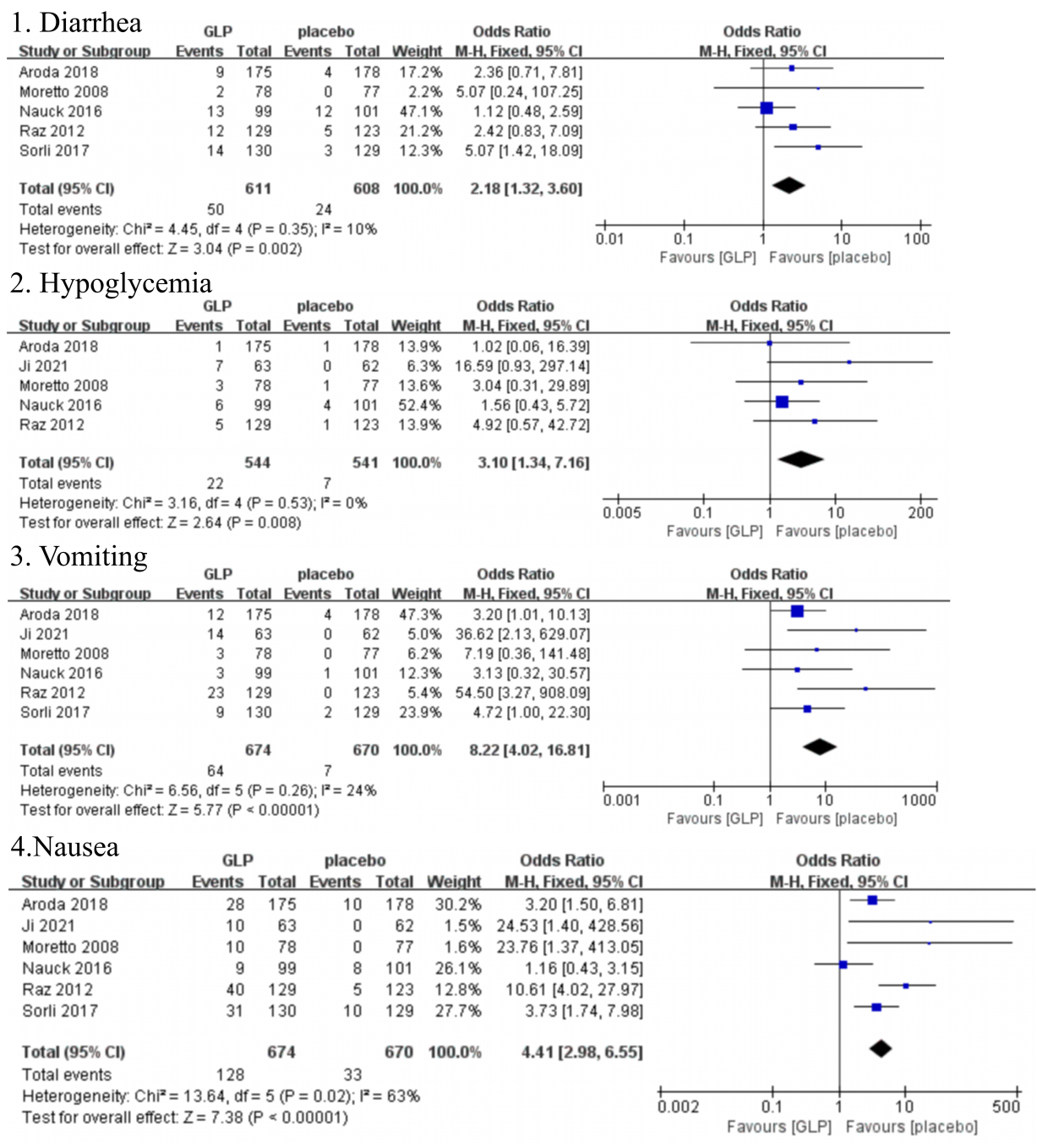
**

1. **Forest plot results of safety outcomes for SGLT2i compared to the placebo group. (B) Forest plot results of safety outcomes for GLP-1RAs compared to the placebo group. The diamond indicates the pooled estimate. Red boxes are relative to study size, and the black vertical lines indicate the 95% CIs around the effect size estimate.**

**Abbreviation: SGLT2i: sodium-glucose cotransporter 2 inhibitors. GLP-1RAs: glucagon-like peptide-1 receptor agonists. UTI: urinary tract infection.**

**Figure S2: Cochrane Risk of Bias version 2 Assessment**

**
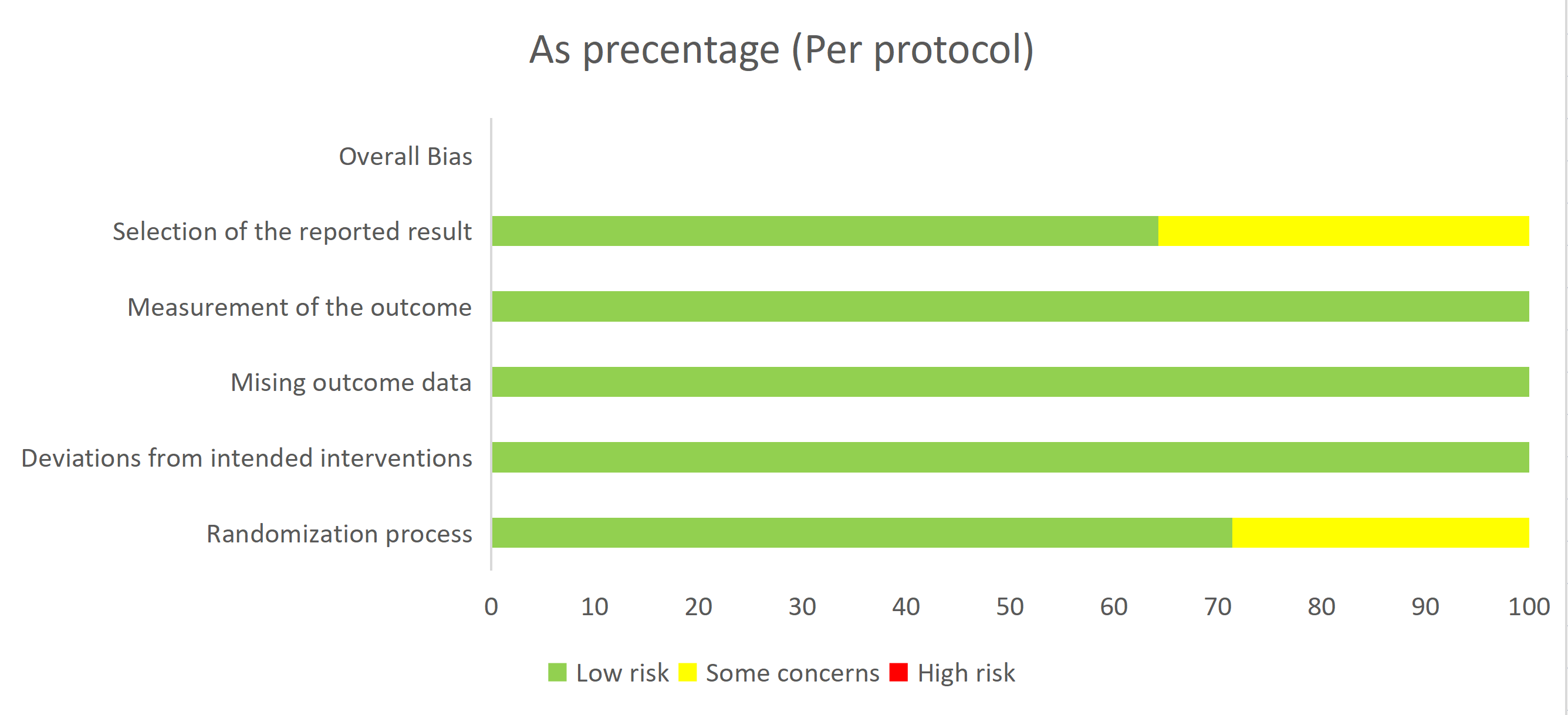
 A**

**B**


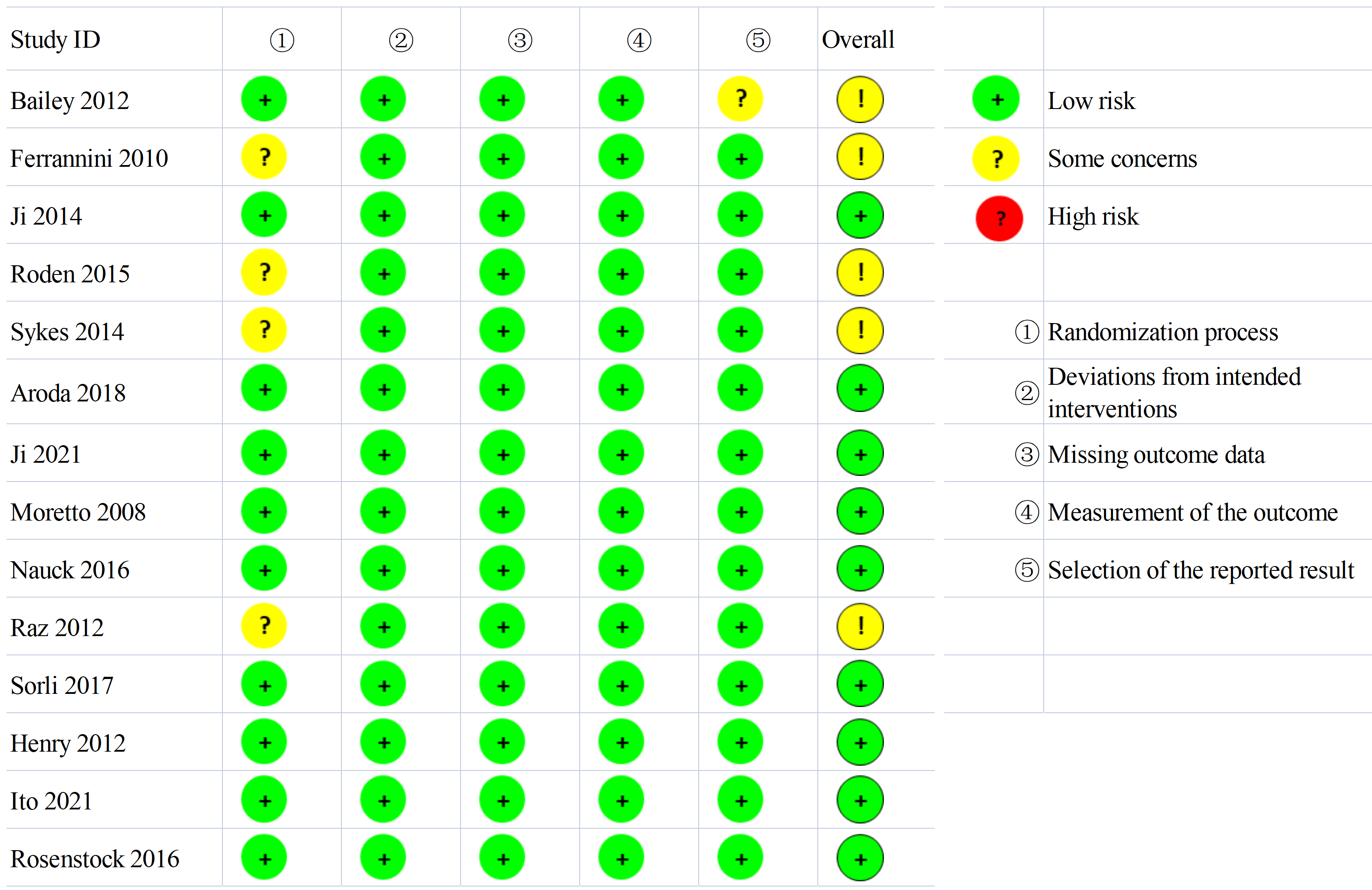

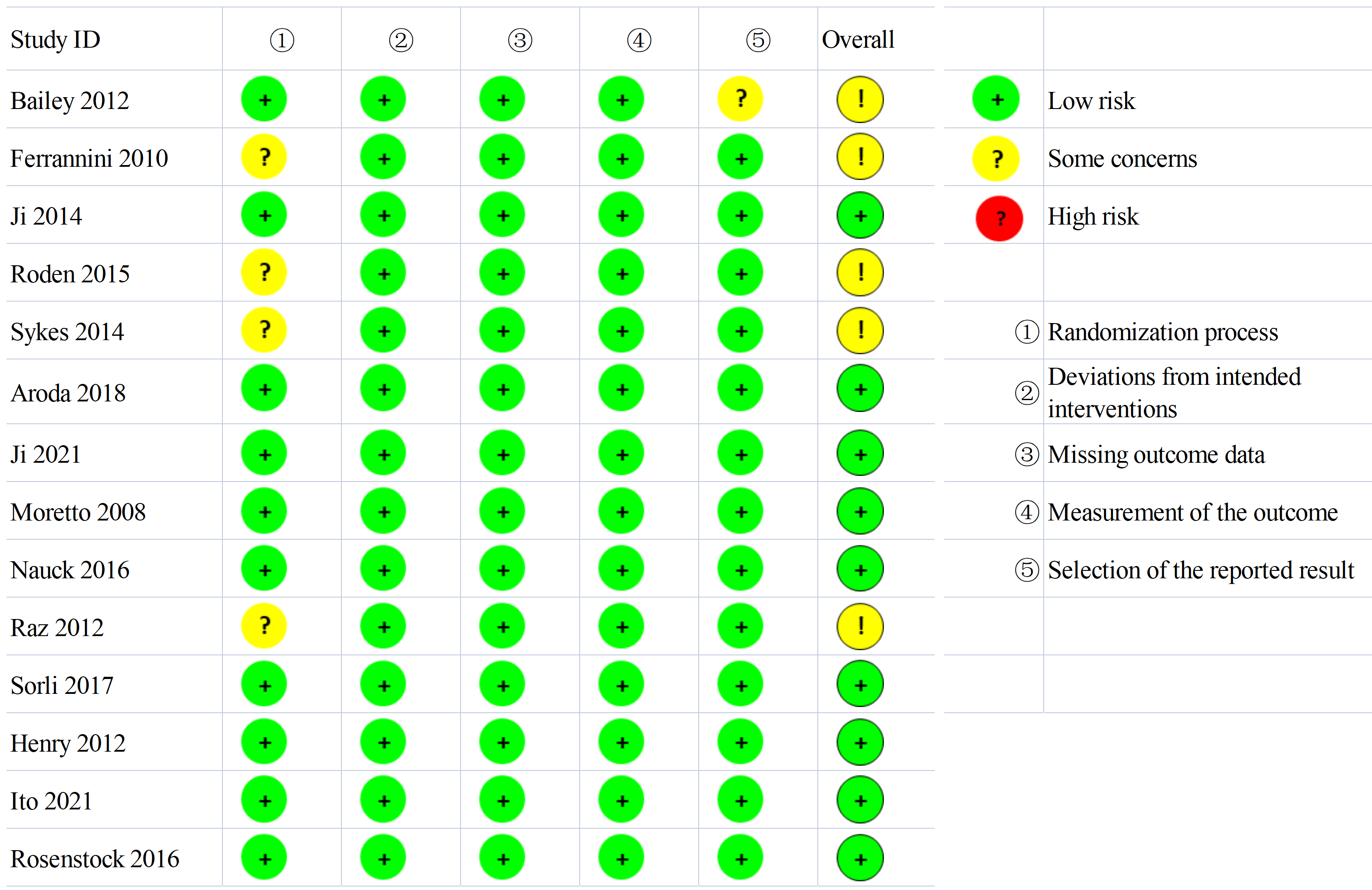


1. **Risk of Bias summary for included studies which applied per-protocol analysis. (B) Risk of bias graph showing a review of authors’ judgements on each risk of bias item.**
